# Expression profiles of respiratory V-ATPase and calprotectin in SARS-CoV-2 infection

**DOI:** 10.1038/s41420-022-01158-3

**Published:** 2022-08-16

**Authors:** Yapeng Hou, Tingyu Wang, Yan Ding, Tong Yu, Yong Cui, Hongguang Nie

**Affiliations:** 1 Department of Stem Cells and Regenerative Medicine, College of Basic Medical Science, China Medical University, Shenyang, 110122 China; 2 Department of Anesthesiology, the First Hospital of China Medical University, Shenyang, 110001 China

**Keywords:** Viral infection, Respiration

## Abstract

Coronavirus disease 2019 (COVID-19) caused by severe acute respiratory syndrome coronavirus 2 (SARS-CoV-2) represents a pandemic threat that has been declared a public health emergency of international concern, whereas the effects of cellular microenvironment in the pathogenesis of SARS-CoV-2 are poorly understood. The detailed message of intracellular/lysosome pH was rarely concerned in SARS-CoV-2 infection, which was crucial for the cleavage of SARS-CoV-2 spike (S) protein. Calprotectin, an endogenous danger signal to activate inflammatory response, was vital for the proceeding of COVID-19. We found that the expressions of both vacuolar-ATPase (V-ATPase) and calprotectin (S100A8/S100A9) increased in SARS-CoV-2 infection, by analyzing single-cell RNA sequencing (bronchoalveolar lavage fluid), bulk-RNA sequencing (A549, lung tissue, NHBE), and proteomics (lung tissue), respectively. Furtherly, our wet experiments of flow cytometry and fluorescent assay identified that the intracellular and lysosome pH value was decreased after SARS-CoV-2 S plasmid transfection in A549 cells. Meanwhile, the enhancement of V-ATPase and calprotectin was verified by our real-time polymerase chain reaction and western blot experiment. Collectively, these data suggested that S protein increased V-ATPase in SARS-CoV-2 infection, which provided a microenvironment easier for the cleavage of S protein, and inflammatory cells were apt to be activated by the enhancement of calprotectin in respiratory epithelium. The comprehensive information on profiles of V-ATPase and calprotectin will make clearer about the involvement of cellular microenvironment in the pathogenesis of SARS-CoV-2, and provide a promising approach to combat COVID-19.

## Introduction

Coronavirus disease 2019 (COVID-19) is a pandemic respiratory infectious disease caused by severe acute respiratory syndrome coronavirus 2 (SARS-CoV-2). Currently, various therapeutic agents such as the antibody drugs, and polymerase/protease inhibitors have been evaluated, but the pathogenesis of SARS-CoV-2 is still not fully understood [1–3]. Compared with other coronavirus, SARS-CoV-2 has higher rates of transmissibility due to the insertion of furin site in spike (S) protein [4–6]. It has been reported that the acidic microenvironment is mainly created by vacuolar-ATPase (V-ATPase), which is necessary for furin to be active in cellular lysosomes, as well as fusion process of SARS-CoV-2 and host membranes [7–9]. Recent genome-wide CRISPR-Cas9 screens have identified V-ATPase subunits as SARS-CoV-2 cofactors [10–12], among which V1 peripheral domain is composed of 8 subunits (A-H), which drives ATP hydrolysis to energize and triggers the rotation of the V0 membrane domain, whereas the latter is composed of a, c, c˝, d, and e subunits, which utilizes the energy generated by the V1 domain to translocate protons across the membrane [13, 14]. As for the involvement of innate immune system in SARS-CoV-2 infection, calprotectin (S100A8/S100A9) has been identified as an important endogenous alarmin, which regulates lymphocyte, monocyte, and neutrophil migration [15]. The high levels of calprotectin in chronic inflammatory pathologies suggest that S100A8/S100A9 might play a role in inflammatory reactions, including SARS infection [16].

Contrary to the highly concerned expression level of calprotectin in immune cells, its role in respiratory epithelial cells was rarely considered [17]. Of note, therapies that focus on correcting the intra- and extra-cellular pH and calprotectin expression may benefit the severe COVID-19 patients. Our study shows for the first time how the acidified microenvironment format and local immune system respond to SARS-CoV-2 infection, which will open possibilities for designing new diagnostics and treatments for this new life-threatening disease.

## Results

### Single-cell RNA sequencing of bronchoalveolar lavage fluid cells in COVID-19 patients

**Fig. 1:**
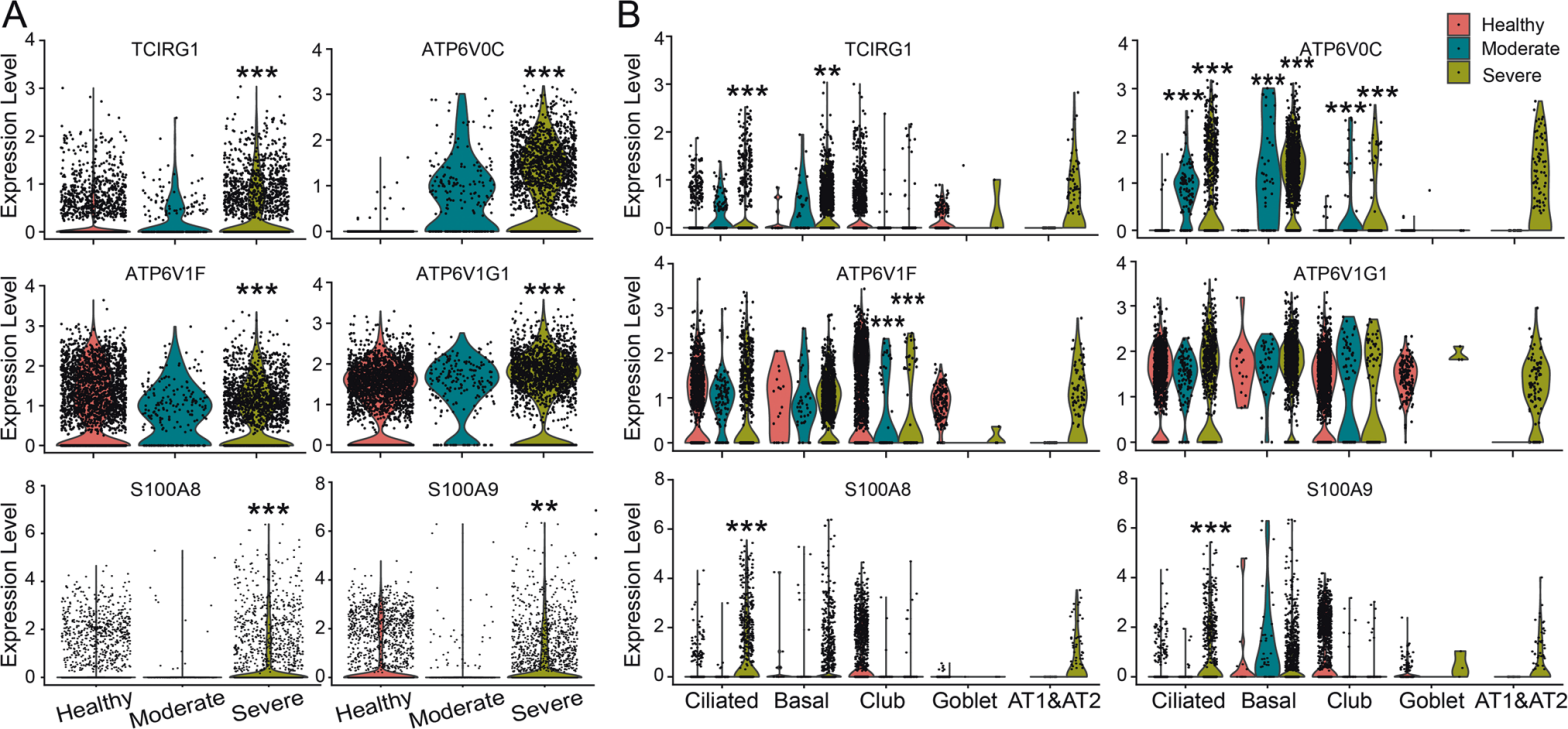
scRNA sequencing analysis of V-ATPase and calprotectin in BALF of COVID-19 patients. **A** Expression level of significantly changed V-ATPase subunits and calprotectin in total epithelial cells of Control, Moderate and Severe COVID-19 cases. **B** Expression level of V-ATPase subunits and calprotectin in ciliated, basal, goblet, club, and alveolar type 1 and type 2 cells (AT1&AT2) of Control, Moderate and Severe COVID-19 cases. ^**^P < 0.01, ^***^P < 0.001, compared with Control group.

The V-ATPase resides within many intracellular compartments, and functions to acidify intracellular compartments [9]. Meanwhile, calprotectin (S100A8/S100A9) is an endogenous danger signal, which can activate inflammatory response [15]. According to our single-cell RNA (scRNA) sequencing analysis, both V0 membrane domain (a3 and c subunits) and V1 peripheral domain (F and G1 subunits) of V-ATPase, and S100A8/S100A9 were all significantly upregulated in total epithelial cells from bronchoalveolar lavage fluid (BALF) of severe COVID-19 patients (Fig. 1A). Furtherly, we analyzed the expression levels of significantly changed genes in different respiratory epithelial cell types, which showed that TCIRG1 (a3 subunit) was significantly upregulated in ciliated and basal cells, whereas the upregulation of S100A8 and S100A9 occurred in ciliated cells of severe COVID-19 cases. Similarly, the ATP6V0C (c subunit) and ATP6V1F (F subunit) of V-ATPase also showed cell type-specific expression levels in moderate/severe COVID-19 patients (Fig. 1B).

### Upregulation of V-ATPase and calprotectin at protein/transcription level

**Fig. 2:**
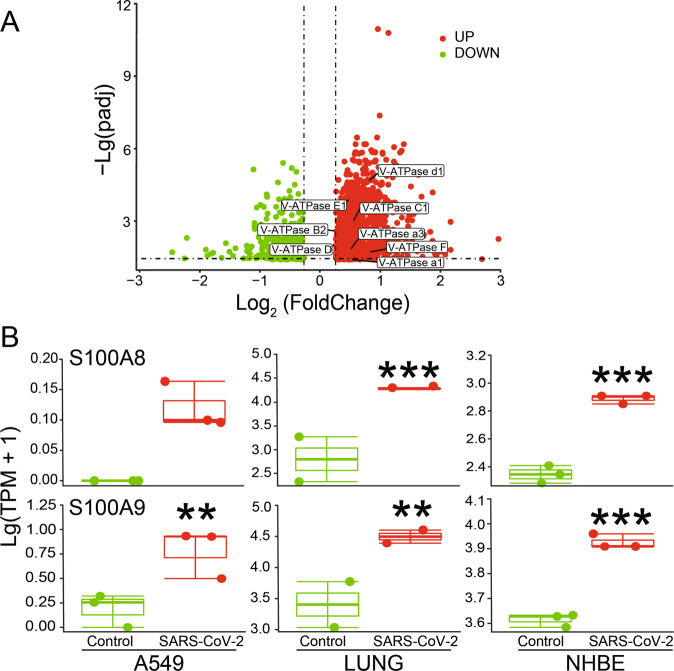
Proteomics and bulk-RNA sequencing analysis after SARS-CoV-2 infection. **A** Proteomics data of lung tissue from COVID-19 patients. The *P* value was corrected with Benjamini-Hochberg correction. Specially tagged V-ATPase subunits were considered significant, due to the adjusted *P* (padj) < 0.05 and Log_2_(FoldChange) > 1.2. **B** Bulk-RNA sequencing analysis of SARS-CoV-2 infected A549 (*n* = 3), lung tissue (*n* = 2), and NHBE cells (*n* = 3). ^**^P < 0.01, ^***^P < 0.001, compared with Control group.

The proteomics data showed that in lung tissues of COVID-19 patients, the protein expression levels of 8 V-ATPase subunits, including 3 V0 membrane domain subunits (a1, a3, and d1) and 5 V1 peripheral domain subunits (B2, C1, D, E1, and F), were significantly upregulated (Fig. 2A). Due to the scarcity of proteomics data for S100A8/S100A9, we analyzed the corresponding bulk-RNA sequencing data accordingly. As expected, S100A8 and S100A9 were both significantly upregulated in SARS-CoV-2 infected A549 cells, human lung tissue, and NHBE cells (Fig. 2B).

### Decrease of intracellular/lysosome pH by SARS-CoV-2 spike protein in A549 cells

**Fig. 3:**
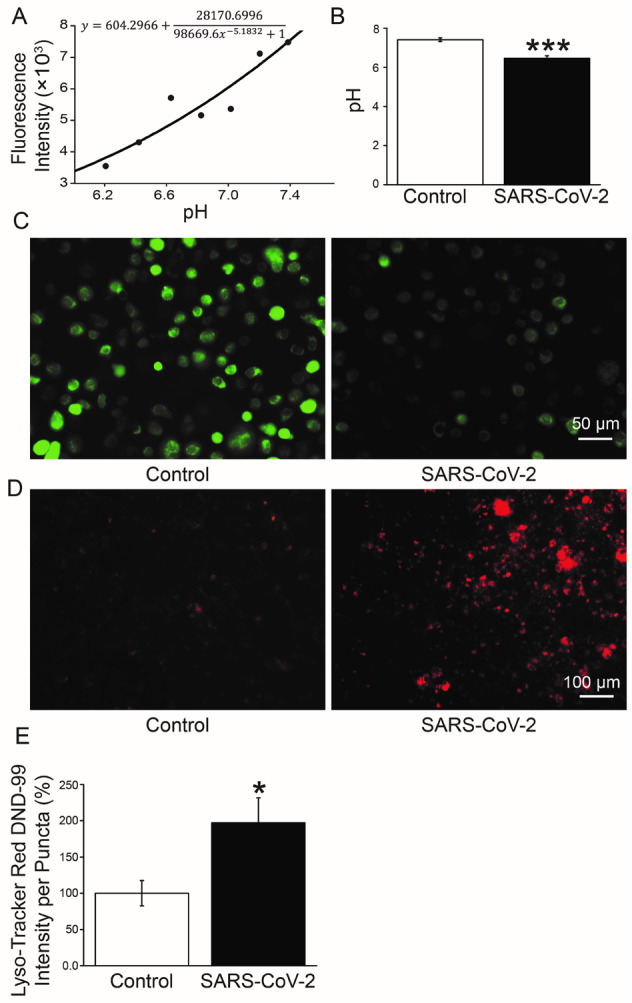
Measurement of intracellular/lysosome pH in A549 cells. **A** The standard curve of fluorescence intensity of BCECF and intracellular pH value by flow cytometry. **B** pH values of A549 cells in Control and SARS-CoV-2 group. ^***^P < 0.001, compared with Control. *n* = 6, one-way ANOVA followed by Bonferroni’s test was used to analyze the difference of the means. **C** Fluorescent intensity of BCECF. **D** Lyso-Tracker Red DND-99 staining. **E** Mean Lyso-Tracker Red DND-99 fluorescence intensity per punctum in A549 cells. *n* = 5, Mann–Whitney *U* test was used to analyze the difference of the means for significance.

Based on the above analysis, we speculate that the enhanced V-ATPase expression may alter the intracellular/lysosome pH in SARS-CoV-2 infection. The standard curve of fluorescence intensity and intracellular pH value was shown in Fig. 3A. According to the fitted formula, the intracellular pH of A549 cells in Control (transfected with pCMV14-3X-Flag) and SARS-CoV-2 group (transfected with pCMV14-3X-Flag-SARS-CoV-2 S plasmid) was about 7.41 and 6.46, respectively (Fig. 3B, *P* < 0.001, *n* = 4–6), consistent with the obviously weaker fluorescent intensity of BCECF after SARS-CoV-2 S plasmid transfection (Fig. 3C). To furtherly identify the pH changes at subcellular level, we applied a cell-permeable weak base dye that was acidotropic and accumulated in acidified organelles, LysoTracker Red DND-99, to determine the changes in lysosomes. The fluorescent images showed that the intensity was obviously strengthened in S plasmid transfected group, supporting the evidence that the lysosomes were acidified by S protein (Fig. 3D, E).

### Enhancement of V-ATPase and calprotectin mRNA expression mediated by SARS-CoV-2

**Fig. 4:**
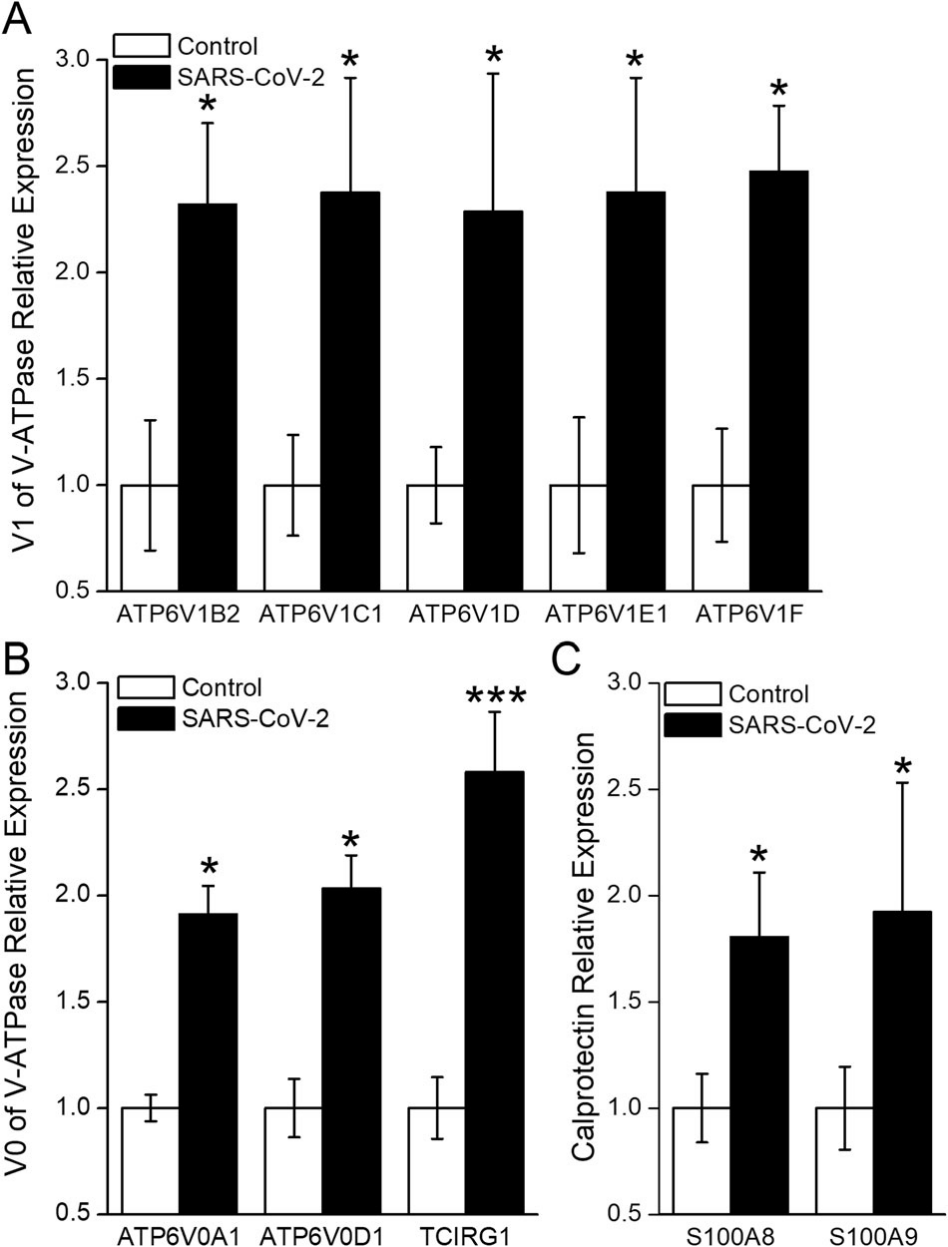
mRNA expression level of V-ATPase and calprotectin. **A**, **B** mRNA expression of 5 V1 peripheral domain subunits: B2 (ATP6V1B2), C1 (ATP6V1C1), D (ATP6V1D), E1 (ATP6V1E1), and F (ATP6V1F); and 3 V0 membrane domain subunits: a1 (ATP6V0A1), a3 (TCIRG1), and d1 (ATP6V0D1). **C** RNA expression level of S100A8 and S100A9. ^*^P < 0.05, ^***^P < 0.001, compared with Control. *n* = 3–5, student’s *t*-test was used to analyze the difference of the means for significance.

To validate the above bioinformatic analysis of V-ATPase and calprotectin, we performed real-time polymerase chain reaction (PCR) to measure the mRNA expressions of significantly upregulated V-ATPase subunits, S100A8, and S100A9, respectively. As shown in Fig. 4A, B, the 5 V1 peripheral domain subunits: B2 (ATP6V1B2), C1 (ATP6V1C1), D (ATP6V1D), E1 (ATP6V1E1), and F (ATP6V1F); and 3 V0 membrane domain subunits: a1 (ATP6V0A1), a3 (TCIRG1), and d1 (ATP6V0D1) were all upregulated significantly in SARS-CoV-2 S plasmid transfected A549 cells (*P* < 0.001–0.05). Coincidentally, the mRNA expression of S100A8 and S100A9 was also enhanced by SARS-CoV-2 S protein (Fig. 4C, *P* < 0.05, *n* = 3–5).

### Increased V-ATPase and calprotectin protein expression after spike plasmid transfection

**Fig. 5:**
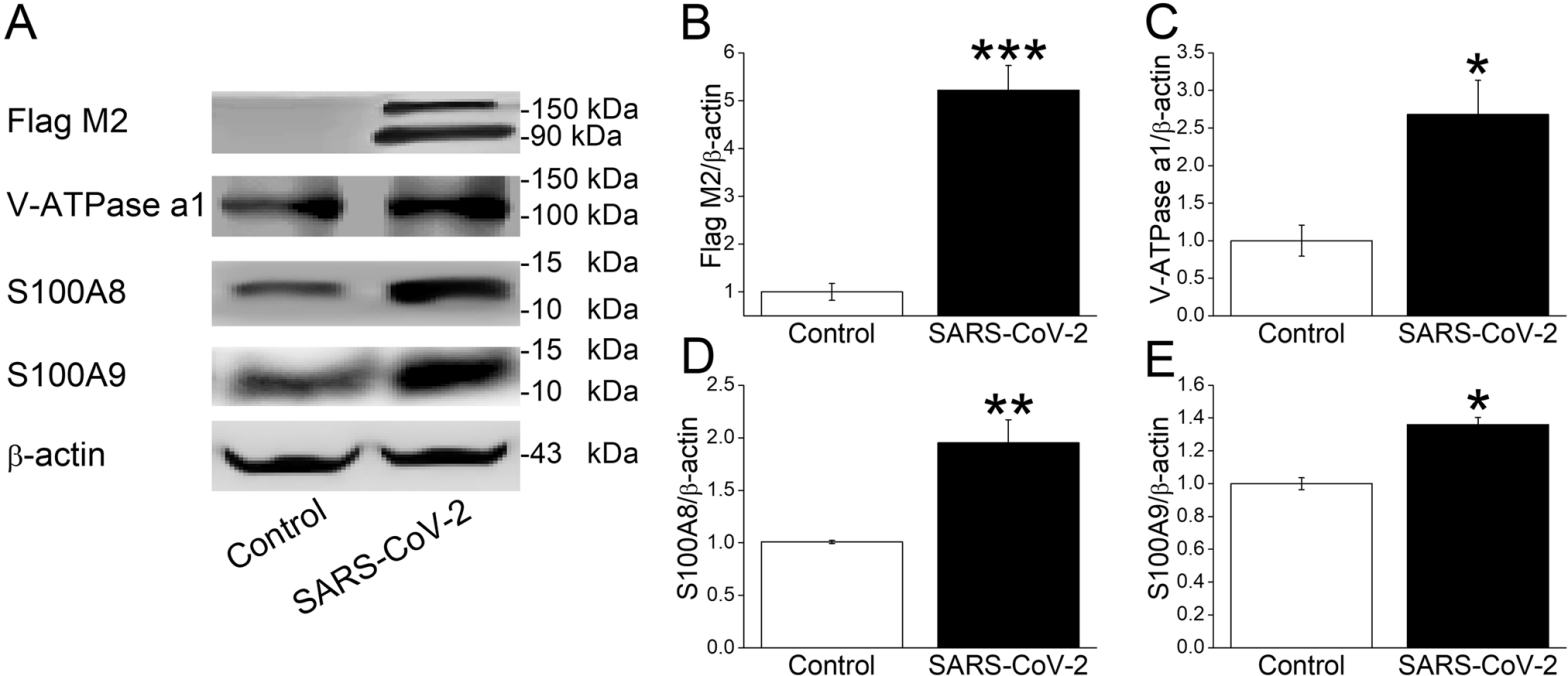
Protein expression of V-ATPase and calprotectin. **A** Representative blots of Flag M2, V-ATPase a1 subunit, S100A8, and S100A9 after SARS-CoV-2 S plasmid transfection. **B**–**E** The statistical data. ^*^P < 0.05, ^**^P < 0.01, ^***^P < 0.001, compared with Control group. *n* = 3–4, student’s *t*-test was used to analyze the difference of the means for significance.

Upregulation of V-ATPase, S100A8, and S100A9 at protein level was further verified by western blot assay. S protein of SARS-CoV-2 was first identified by the 180 kDa and 90 kDa (Flag M2) bands after plasmid transfection in A549 cells, whereas there were no corresponding bands in Control group (Fig. 5A, B, *P* < 0.001, *n* = 4) [18]. Considering its important role in intracellular/lysosome pH regulation, the a1 subunit of V-ATPase was measured by western blot, which showed significant upregulation in SARS-CoV-2 group (Fig. 5A, C, *P* < 0.05) [19]. Consistent with previous real-time PCR and RNA sequencing data, S100A8 and S100A9 were also significantly increased at protein level after SARS-CoV-2 S plasmid transfection (Fig. 5A, D, E, *P* < 0.01–0.05, *n* = 3).

## Discussion

**Fig. 6:**
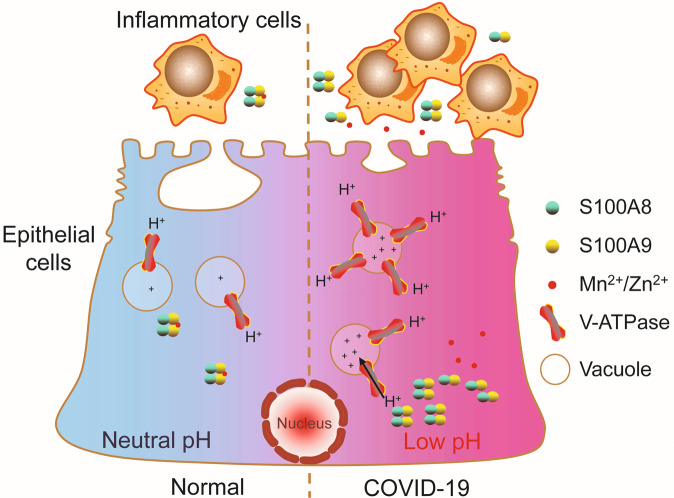
The schematic diagram of intracellular pH dysregulation during SARS-CoV-2 infection. SARS-CoV-2 increased the expression level of V-ATPase and calprotectin (S100A8/S100A9) in respiratory epithelial cells. The increased V-ATPase provided an acidic microenvironment easier for the cleavage of S protein, whereas the sequester ability of calprotectin with metal ions (Mn^2+^/Zn^2+^) was reduced in acidic pH, triggering the consequent inflammation response of respiratory epithelium.

In this study, we reported that SARS-CoV-2 decreased the intracellular/lysosome pH by increasing the expression of V-ATPase, and was prone to activating inflammatory cells by the enhancement of calprotectin (Fig. 6). One potential advantage in our current data analysis was that the gene expression levels were compared between patient samples and normal controls, using both total respiratory epithelial cells and individual cell types. The observations reported here highlighted the importance of ciliated cells in pulmonary acid-base regulation and the critical role of the V-ATPase in the function of these fascinating cells. This differential gene expression profiling of calprotectin in epithelial cells from COVID-19 cases supported that the response of affected patients to SARS-CoV-2 seemed mainly to be an innate inflammatory one [16, 20].

The physiological stable pH around 7.4 is critical for the dependence of proteins that are charged macromolecules, abnormal regulation of which would impair many cell functions [21]. Intra- and extra-cellular pH may influence the entry of SARS-CoV-2, and Niclosamide, which can change the intracellular pH value has been considered as a candidate for COVID-19 treatment [7, 22]. S protein could decrease the intracellular/lysosome pH value in our wet experiment, whereas had no obvious effects on extracellular pH, possibly for the powerful buffer system in culture medium (data not shown). A recent publication reported that SARS-CoV-2 deacidified the lysosomes to egress the virion, which was mediated by open reading frame protein 3 A [23]. The discrepancy of our results with the above may be due to the different stages during virus processing. We hypothesize that at the early stage of virus synthesis and translocation to the lysosome, the acidic microenvironment may be beneficial for the cleavage of furin site by proteases. Whereas at the later stage after the cleavage was done, the deacidification of lysosome by open reading frame protein 3 A would make the virion easier to egress.

V-ATPase is closely related to the acidification of urine or gastric juice, macrophages, neutrophils, and so on. Meanwhile, small airways are acidified by V-ATPase, which is identified as a novel therapeutic target for small airway related diseases and drugs targeting V-ATPase have been suggested for COVID-19 treatment [24]. Considering the S protein was able to induce pulmonary infectious diseases and the limitation of virus biosafety, we transfected S plasmid into A549 cell to mimic the acute lung injury during SARS-CoV-2 infection [25]. We speculate that the enhanced V-ATPase expression would acidify the intra-cellular microenvironment necessary for the virus processing [26], supported by the evidence that the V-ATPase inhibitor Bafilomycin A1 could suppress SARS-CoV-2 entry/replication owning to that the deacidified lysosome could not provide a suitable microenvironment for the cleavage of S protein [18, 24].

Calprotectin, which was found in the extracellular milieu during infections and inflammatory episodes, has been identified as an important endogenous alarmin, one of the damage-associated molecular pattern molecules that acts as a ligand for the Toll-like receptor 4 receptor and amplifies the inflammation cascade *via* NF-κB and p38 mitogen-activated protein kinase [15]. The enhancement of calprotectin is apt to activate inflammatory cells, supporting the involvement of innate immune response in SARS-CoV-2 infection [16, 20].

For the destroyed innate immune system by SARS-CoV-2, COVID-19 patients are prone to bacterial infections, which are considered critical risk factors for the severity and mortality rates [27]. Two host-defense defects have been reported in gland-containing airways, including the mucociliary transport and bacterial killing defect in acidic airway surface liquid [26]. Calprotectin, a heterodimeric complex of calcium-binding proteins S100A8 and S100A9, protects the airway epithelium from pathogens by sequestering the transition metal ions (including Mn^2+^/Zn^2+^) in the extracellular space to limit nutrient availability and the growth of invading microbial pathogens under neutral pH environment, while the sequester ability of calprotectin with metal ions was reduced in acidic pH (Fig. 6) [28–30].

Due to the association with clinical outcomes such as significantly reduced survival time, calprotectin represents an intriguing and promising biomarker for COVID-19 severity [20]. Analysis of human strains expressing V-ATPase and calprotectin will provide valuable information to understand the molecular mechanisms leading to the inclined virus invasion and increased inflammatory response in SARS-CoV-2 infection, and open up novel avenues for treatment of COVID-19 patients.

## Materials and methods

### scRNA sequencing and proteomics data analysis

scRNA sequencing data of cells from BALF were download from Gene Expression Omnibus by using the accession number GSE122960 and GSM3660650. The BALF cells were obtained from four healthy Control, three moderate, and six severe COVID-19 patients. The COVID-19 patient classification and demographic characteristic of donors studied by scRNA-seq/proteomics were reported in previous study [31, 32]. The scRNA-seq data were processed with the code published in Github (https://github.com/zhangzlab/covid_balf) with Seurat V3.2.3 [31].

After the filtration, dimension reduction, and clustering, we compared gene expression levels in total epithelial cells and different epithelial cells types by using the non-parametric hypothesis test (Wilcox-test) among healthy Control, Moderate and Severe COVID-19 group, respectively [31].

We analyzed the proteomics from the supplementary files of previous publication [32]. The data of human lung tissue were composed of 6 healthy Control and 15 COVID-19 specimens. Log_2_(FoldChange) indicated the ratio of protein expression in lung tissues between Control and COVID-19 group. The *P* adjust (padj) < 0.05 and the Log_2_(FoldChange) > 1.2 was considered significant. The volcano plot and boxplot were graphed by ggplot2 (version 3.3.2).

### Bulk RNA sequencing

The bulk RNA sequencing data were downloaded from Gene Expression Omnibus database (Accession number: GSE147507). The dataset contained the RNA sequencing data of SARS-CoV-2 (USA-WA1/2020) infected primary human bronchial epithelium (NHBE), human alveolar epithelial cells (A549), and human lung tissue. Differently expressed genes between Control and SARS-CoV-2 group were determined by DESeq2 (Version 1.26.0) with Wald test and Benjamini-Hochberg post-hoc test in A549 cells, human lung tissue, and NHBE cells, respectively [33, 34].

### Cell culture and transfection

A549 cells purchased from American Type Culture Collection were cultured in six-well plate with RPMI 1640 medium, containing 10% fetal bovine serum and 1% penicillin-streptomycin. To figure out the expression of S protein of SARS-CoV-2, A549 cells were randomly transfected 2 μg pCMV14-3X-Flag-SARS-CoV-2 S plasmid (a gift from Zhaohui Qian, Addgene, Cat.Num:145780; http://n2t.net/addgene:145780; RRID: Addgene_145780) with 4 μl polyetherimide (1 mg/ml, Yeasen, Shanghai, China), and protein expression was determined 48 h post transfection [18]. The cells in Control group were transfected with pCMV14-3X-Flag (purchased from Public Protein/Plasmid Library).

### Intracellular/lysosome pH measurement

The intracellular pH was measured according to previous studies [35, 36]. Briefly, A549 cells were incubated with BCECF-AM (5 μM, Solarbio, Beijing, China) for 1 h in 5% CO_2_ incubator, to establish the standard curve of fluorescent intensity and pH values. The cells were treated with solution containing nigericin (5 μM, dissolved in ethanol, Yuanye, Shanghai, China) at different pH values (6.2, 6.4, 6.6, 6.8, 7.0, 7.2, and 7.4) for 15 min, respectively. After the A549 cells were labeled with BCECF-AM, the fluorescent intensity was measured by flow cytometry. The Four Parameter Logistic Curve Calculator was used to fit the equation for fluorescent intensity and pH value. Meanwhile, the fluorescent intensity of BCECF was also observed with fluorescent microscope.

A549 cells were incubated with 75 nM Lyso-Tracker Red DND-99 (Yeasen, Shanghai, Chian) for 45 min according to manufacturer instructions. To determine the intensity of Lyso-Tracker Red DND-99 per puncta, five images per well were randomly collected by fluorescent microscope.

### Real-time PCR

The total RNA in A549 cells was extracted by TRIzol reagent (Invitrogen, Waltham, MA, United States), and 500 ng RNA was used as template to synthesize cDNA with reverse transcription kit (TaKaRa, Kusatsu, Japan) after the concentration was measured by spectrophotometry. The real-time PCR was performed by the following program: a single cycle of 95 °C for 0.5 min, 40 cycles of 95 °C for 5 s, and 60 °C for 34 s in the ABI 7500 real-time PCR System. All primers were obtained from PrimerBank (https://pga.mgh.harvard.edu/primerbank/index.html) and listed in Supplementary Table 1. Relative expression of mRNA was calculated by 2^−ΔΔCT^, and *Actb* (β-actin) was used as an internal reference.

### Western blot assay

The A549 cells were lysed by laemmli buffer for 20 min at 4 °C. Extracted proteins were separated on SDS-PAGE, and transblotted onto PVDF membranes. The blots were incubated with primary antibody overnight after blocked with 5% bovine serum albumin for 1 h. The secondary antibody was incubated with the membranes for 1 h, then the images were developed by ECL kit (Tanon, Shanghai, China) and analyzed with Image J. The information of antibodies was listed in Supplementary Table 2. The full length uncropped original western blots were showed in Supplementary Fig. 1.

### Statistical analysis

The power of sample size was first evaluated to meet *P* < 0.05. Student’s *t*-test or one-way analysis of variance (ANOVA) followed by Bonferroni’s test was applied for comparing the differences of means between groups, if the data passed the normality (Shapiro–Wilk) and homoscedasticity (Levene) test. Otherwise, non-parametric *t*-test (Mann–Whitney *U*-test) was used. Statistical analysis was performed with Origin 8.0. All the data were collected by the investigators, who were blinded to the treatment of the cells.

## Supplementary information

Supplementary file

## Data Availability

All data generated or analyzed during this study are available from the corresponding author on reasonable request.

## Code availability

All code generated or analyzed during this study are available from the corresponding author on reasonable request.
