# Supplementary material for: Expression profiles of respiratory V-ATPase and calprotectin in SARS-CoV-2 infection
Source: Cell Death Discov. 2022 Aug 16;8:362. doi: 10.1038/s41420-022-01158-3 (PMC9379883; doi:10.1038/s41420-022-01158-3)
Supplement: Supplementary file 1 — Supplementary file [file 41420_2022_1158_MOESM1_ESM.docx]

**Supplementary information**

**Expression** **profiles of respiratory V-ATPase and calprotectin in SARS-CoV-2 infection**

Yapeng Hou ^1^, Tingyu Wang ^1^, Yan Ding ^1^, Tong Yu ^1^, Yong Cui ^2^, Hongguang Nie ^1,^ *

*^1^ Department of Stem Cells and Regenerative Medicine, College of Basic Medical Science, China Medical University, Shenyang, 110122, China*

*^2^ Department of Anesthesiology, the First Hospital of China Medical University, Shenyang, 110001, China*

Running Title: Profiles of V-ATPase/calprotectin in COVID-19

* Corresponding author.

Email address: hgnie@cmu.edu.cn (H. Nie)

**Supplementary Table 1.** Primers for real-time PCR

| Protein | Gene | Forward (5'-3') | Reverse (5'-3') |
| --- | --- | --- | --- |
| V-ATPase 116 kDa subunit a1 | ATP6V0A1 | GGGAGCGCATCCCTACTTTT | GGTTCTCGATTTCAGCCTGTC |
| V-ATPase subunit d1 | ATP6V0D1 | TTCCCGGAGCTTTACTTTAACG | CAAGTCCTCTAGCGTCTCGC |
| V-ATPase 116 kDa subunit a3 | TCIRG1 | CATGGTCCTTGCGGAGAACC | GCCGGTGTAGATGGAGAACAG |
| V-ATPase subunit B2 | ATP6V1B2 | AGTCAGTCGGAACTACCTCTC | CATCCGGTAAGGTCAAATGGAC |
| V-ATPase subunit C1 | ATP6V1C1 | GAGTTCTGGCTTATATCTGCTCC | GTGCCAACCTTTAAGTCAGGAAT |
| V-ATPase subunit E1 | ATP6V1E1 | AACATAGAGAAAGGTCGGCTTG | GACTTTGAGTCTCGCTTGATTCA |
| V-ATPase subunit F | ATP6V1F | CTCATCGCAGTGATCGGAGAC | CGGTTCTTGTTAAGCTCCCCTAT |
| V-ATPase subunit D | ATP6V1D | AGCACAGACAGGTCGAAACC | TTCTCTCATCACTTCGCCCAT |
| S100A8 | S100A8 | CAACACTGATGGTGCAGTTAACTTC | CTGCCACGCCCATCTTTATC |
| S100A9 | S100A9 | CTGAGCTTCGAGGAGTTCATCA | CGTCACCCTCGTGCATCTTC |

**Supplementary Table 2.** Information about antibodies

| Antibody | Manufacture | Cat.log. number | Dilution |
| --- | --- | --- | --- |
| S100A8 | Proteintech | 15792-1-AP | 1:1000 |
| S100A9 | Proteintech | 26992-1-AP | 1:1000 |
| Flag M2 | Sigma | F3165 | 1:1000 |
| V-ATPase a1 kDa subunit | Santa Cruz | sc-374475 | 1:1000 |
| β-actin | Santa Cruz | sc-47778 | 1:3000 |


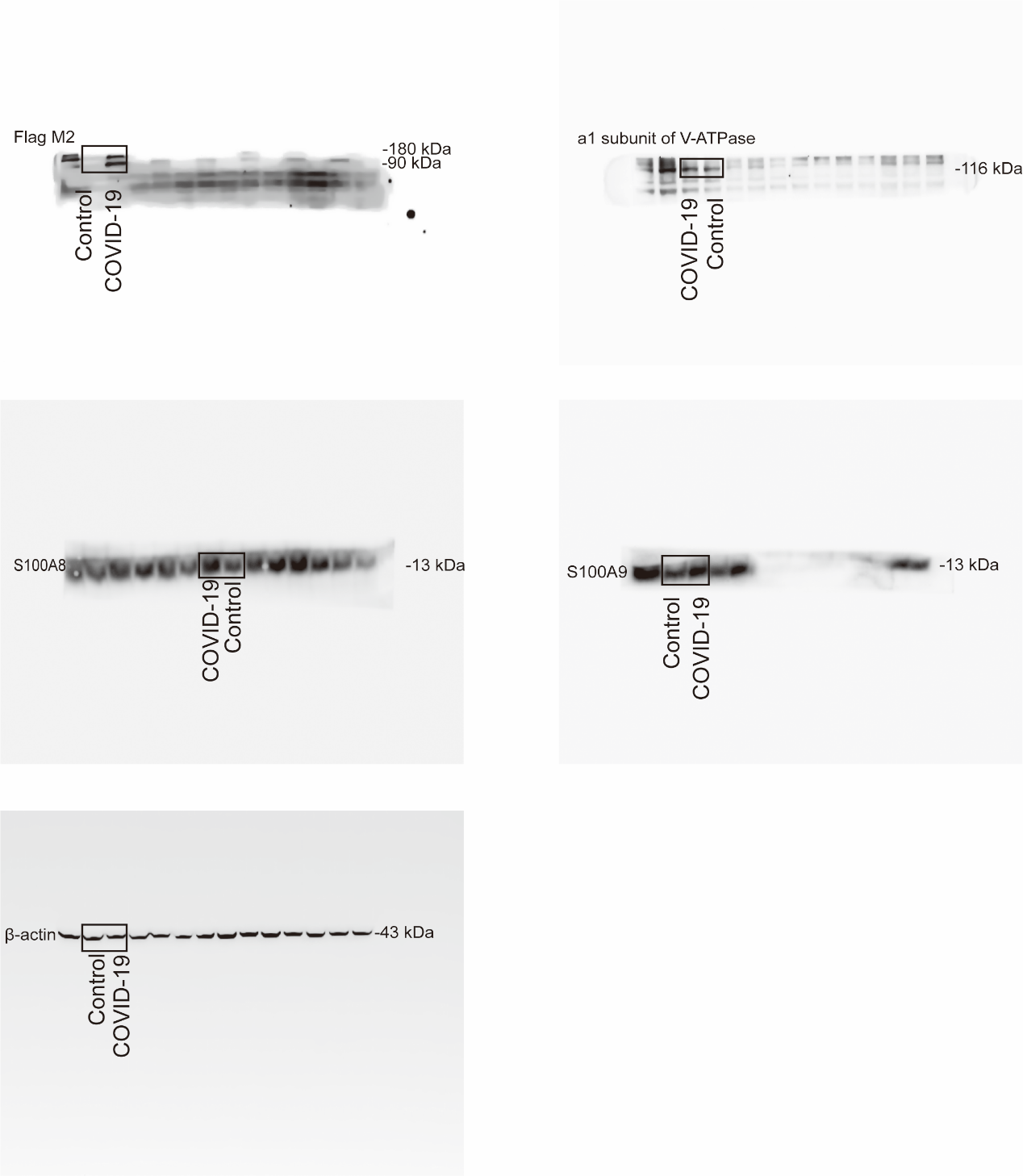


**Supplementary Fig. 1. Full length uncropped original western blots.**
